# Role of Quantum Information in HEOM Trajectories

**DOI:** 10.1021/acs.jctc.4c00144

**Published:** 2024-06-18

**Authors:** Ben S. Humphries, Joshua C. Kinslow, Dale Green, Garth A. Jones

**Affiliations:** † School of Chemistry, University of East Anglia, Norwich Research Park, Norwich NR4 7TJ, U.K.; ‡ Physics, Faculty of Science, University of East Anglia, Norwich Research Park, Norwich NR4 7TJ, U.K.

## Abstract

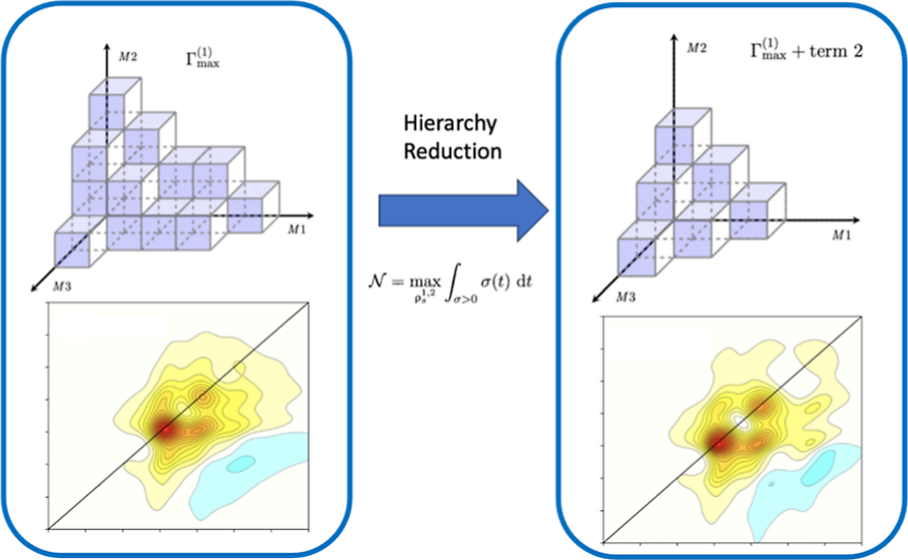

Open quantum systems often operate in the non-Markovian regime where a finite history of a trajectory is intrinsic to its evolution. The degree of non-Markovianity for a trajectory may be measured in terms of the amount of information flowing from the bath back into the system. In this study, we consider how information flows through the auxiliary density operators (ADOs) in the hierarchical equations of motion. We consider three cases for a range of baths, underdamped, intermediate, and overdamped. By understanding how information flows, we are able to determine the relative importance of different ADOs within the hierarchy. We show that ADOs sharing a common Matsubara axis behave similarly, while ADOs on different Matsubara axes behave differently. Using this knowledge, we are able to truncate hierarchies significantly, thus reducing the computation time, while obtaining qualitatively similar results. This is illustrated by comparing 2D electronic spectra for a molecule with an underdamped vibration subsumed into the bath spectral density.

## 1 Introduction

Open quantum systems (OQS) are approaches used for theoretically investigating the behavior of quantum particles that are embedded in complex environments. The system of interest is treated quantum-mechanically through a system Hamiltonian, and the environment is generally described by a bath that is composed of an infinite set of harmonic oscillators conforming to a spectral density that describes environmental fluctuations. Interaction between the system and the environment occurs via an interaction Hamiltonian.

Building a realistic OQS requires careful consideration of the structure of the bath and the system–bath boundary. The bath affects the system’s fluctuations and is responsible for dissipation which involves energy transfer from the system to the bath. In some cases, energy may also be transferred from the bath back to the system. In chemical physics, OQS approaches have been of particular importance within models of electronic energy transfer,^1−3^ charge transfer,^4^ and coherence.^5−8^

Broadly speaking, there are two main varieties of OQS dynamics: Markovian and non-Markovian. The memory of a system, with respect to the state of the bath, is formalized through the Markov property which itself is a statement that a stochastic process is memoryless if its evolution is independent of its history. When considering an evolution, the classical Markov property asserts that each state at a particular time depends solely on the previous state in time. This means that any process which evolves following a scheme such that successive steps depend on more than the previous time step (i.e., are dependent on the history of the bath) are termed non-Markovian and do not satisfy the Markov property. However, more formal definitions exist related to the divisibility of quantum dynamical maps.^9,10^ These formal definitions lead to the identification of quantum Markovianity in relation to the flow of information between the system and the bath. In Markovian cases, information flows unidirectionally out of the system and into the bath throughout the entire trajectory. On the other hand, non-Markovian cases include information flow from the bath back into the system, a process referred to as recurrence. We highlight the work of Li et al. for a rigorous overview of different definitions of non-Markovianity.^11^

There have been a number of procedures that have been developed for modeling non-Markovian OQS.^12,13^ In this work we are focused on the hierarchical equations of motion (HEOM),^14−21^ which is a nonperturbative approach derived from path integrals that has been proven to be highly successful in capturing quantum thermal effects in OQS, such as relaxation of a quantum state, by comparison with analytical models.^20^ Recently the HEOM has found use in the dynamics of excitons,^22−27^ studies of electron transfer,^28−34^ and excited state dynamics within the condensed phase.^35−37^

The HEOM is a system of equations that contain, in principle, an infinite number of auxiliary density operators (ADOs) possessing all the information about the system–bath correlations. As the name suggests, one can think of these coupled equations as being members of a hierarchical network. This structure, however, must of course be truncated for practical implementation. There have been a number of procedures implemented for achieving this including various decomposition techniques,^38−44^ basis set optimization,^45^ tensor network analysis, projection techniques, and ADO normalizations.^32,32,46,47^ Other studies have focused on altering the topology of the hierarchy to tailor it to more complicated baths.^15,44^

In this work, we consider the importance of different ADOs within a hierarchy by explicitly considering the flow of information through the elements of the hierarchy. This gives insights into which ADOs are fundamentally more important to the dynamics for a specific bath and paves the way for an alternative robust truncation procedure.

In Section 2, we overview the theory of quantum information and its relationship to ADOs; in Section 3 the specifics of the OQS used are described; and Section 4 presents the results and discussion prior to our conclusions in Section 5.

## 2 Theory

### 2.1 Quantum Information and Non-Markovianity

Quantum information, the complement of entropy, exists within OQSs and can be transferred between the system and the bath via a quantum channel.^9^ In quantum information theory, these channels are operators through which information is transferred and as such are exact descriptions of the evolution of the density matrix.

The Von-Neumann entropy function links the entropy of a bulk material to discretized portions of the quantum information.^48^ In the case of a pure state, there is a maximum in the total knowledge of the system, and each state in the system can be uniquely defined. This will subsequently correspond to zero entropy. On the other hand, if we have a mixed state where the magnitude of information is proportional to the number of distinguishable microstates,^49^ then there will be nonzero amounts of both information and entropy. Analytically, the entropy function takes the form
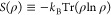
1where ρ is the density matrix for a system describing an ensemble of particles. Based on the previous example, it is clear that the composite entropy function is zero if and only if ρ is a pure state, when evaluated at a specific time through the trace.

The property of Markovianity is numerically explicit and can be further defined through the entropy function and quantum information theory. A Markovian process defines each future state in time based solely on the previous time step, and this corresponds to the entropy functional being concave such that ρ → *S*(ρ), and , for nonvanishing λ_*i*_.^13,48^ Furthermore, when the system is composite, there is a subadditivity condition: H = H^(1)^ ⊗H^(2)^ where *S*(ρ) ≤ *S*(ρ^(1)^) + *S*(ρ^(2)^). Consequently, during a Markovian evolution, the system monotonically loses information to the environment, as each successive state evolves independently of its history toward equilibrium.^9,10,50^ The monotonic loss of information for quantum states is defined by decreasing distinguishability of different quantum systems with time.^51^ In contrast, non-Markovianity corresponds to any situation which invalidates the Markovian condition of monotonic information loss. With reference to the previous example, this could be considered as a mixed state where information about the system in a historical state imprinted onto the environment returns through a quantum channel to influence the future dynamics.

Given these definitions of Markovianity, it is possible to construct a metric on the space of density matrices in order to quantitatively measure the relative distinguishability between two quantum states. This metric is a generalization of the classical Kolmogorov distance and a simplification of the more general Helstrom metric. For a pair of states, ρ_1_ and ρ_2_, the trace distance metric of distinguishability is defined as^50,52,53^
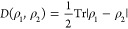
2where . It is important to note that the two density matrices being compared correspond to two different systems at the same time, not the same system at two different times. For example, this may be one density matrix representing a system that has interacted with a laser field and another case where the system has not interacted with that field, as is the case we consider in this work. As discussed in ref (54), this choice of construction ensures that the supports of the kernel matrix are orthogonal when the flux of information is negative, corresponding to an increase in entropy of the system and a loss of distinguishability. The factor of half is omitted in the Helstrom metric and is present here due to the simplifying assumption that each state is equally probable. The maximum and minimum values of the trace distance are observed when the supports are orthogonal or parallel, respectively, which physically correspond to completely distinguishable and completely indistinguishable quantum states.

Given the quantitative distinguishability of quantum states, it is now possible to define a measure of the information flux within the total system. Again, this is usually defined with respect to the system of interest such that a negative information flux is Markovian and a positive information flux is non-Markovian. The information flux^55^ is
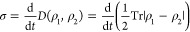
3

Markovianity, in addition to resulting in a concave entropy functional, requires a divisible dynamical map from the time convolutionless master equation, Λ(*t* + τ, 0) = Λ(*t* + τ, *t*)Λ(*t*, 0).^13^ Based on the necessity of a negative flux for a Markovian process, this means that a non-Markovian process must correspond to a strictly positive flux, σ > 0. However, divisibility is not a necessary condition for negative flux. That is to say, a negative flux is a necessary but not sufficient condition for Markovianity. Consequently, we take the information flux for our system and set every negative value to zero, leaving the purely non-Markovian contributions. Then, by integrating over time for the maximum positive flux in the system, we can define a magnitude for the information returned to the system and hence the degree of non-Markovianity. The Breuer–Laine–Piilo (BLP) measure, , has been used previously to quantify the non-Markovianity of a general quantum system in this way^51−53,56,57^
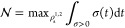
4

Therefore, a non-Markovian evolution has a strictly nonzero information flux and a quantifiable non-Markovianity of . Through this measure, we can relate microscopic non-Markovianity with macroscopic spectral properties.^54^

### 2.2 Virtual Information and ADOs

The HEOM comprise a number of terms, each of which operates on an order of ADO. These auxiliaries are evolved through a series of subequations and define the contributions to the full density matrix. They are structured as an infinite hierarchy, and their positions within that hierarchy dictate their contribution to the density matrix.

The equation of motion for the ADOs of this expansion is then^54^
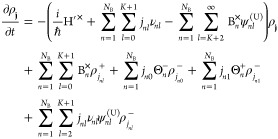
5where
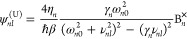
6
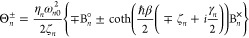
7and B_*n*_^×^ρ = [B_*n*_,ρ] denotes the commutator of the bath coupling operator and the density matrix and B_*n*_^°^ρ = {B_*n*_, ρ} the corresponding anticommutator. The system Hamiltonian is again renormalized to H′. The associated terminator for this hierarchy is^34^
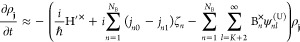
8valid for integers **j** = (*j*_*n*0_,...,*j*_*N*_B_*K*_), with
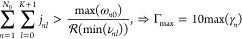
9

In addition, computationally, the sum to infinity is truncated with a sufficiently high value with respect to the criterion Γ_max_.

The terms acting on ρ_**j**_ in eq 5 describe the Markovian free propagation of the system and the impact on this propagation of integer multiples of Matsubara frequencies corresponding to the interaction with bath phonons. By propagating a series of ADOs, representing different arrangements of bath phonons, the HEOM accounts for a history of interactions such that non-Markovian effects are automatically included. The ADOs are interconnected via raising and lowering terms which are denoted by **j**^±^ = (*j*_10_,...,*j*_*nl*_ ± 1,...,*j*_*N*_B_*K*_) vectors. The ρ^–^-dependent terms are raising operations. The action of  and its conjugate is to destroy bath phonons, of coupling amplitude *d*_*nl*_, as they are absorbed by the system. This corresponds to an increase of the ADO tier resulting in a “raising” of the ADO number along a Matsubara axis. Subsequently, this process is associated with thermal fluctuations and the real part of the correlation function because of its temperature dependence. The final term, dependent on ρ^+^, is the corresponding lowering term. The action of  is to destroy the system states, corresponding to the creation of bath phonons as they are emitted from the system into the bath. Destruction of system states in this manner is a consequence of the imaginary part of the correlation function associated with system dissipation. In this way, non-Markovian feedback can occur between the system and bath via the ADOs. An abstract volume can be deduced from the hierarchy structure, which while in principle is infinite becomes finite by the termination of the ADOs based on Markovianity constraints, eq 9. This constraint seals the hierarchy volume. Those terms which include the operator ψ_*nl*_^(U)^ (6) relate to the truncation of the hierarchy and are equivalent to a cumulant expansion treatment of the order of the first neglected term in the truncation scheme, thereby applying a partial ordering prescription.^58^ The application of the truncation scheme, eq 9, therefore constitutes a nonsecular Redfield treatment when more than one electronic level is included in the excited state. The final term in eq 5 is a sum over all thermal Matsubara frequencies and relates to the degree of damping in an underdamped mode. This creates an hierarchy structure where each level couples to the levels above and below. Terminators are necessarily Markovian. Those ADOs which we do not terminate can be either Markovian or non-Markovian (in their virtual information content) based on the physical system parameters.

The entire hierarchy structure defines the nature of the system–bath interaction, and therefore we attribute a degree of physical meaning to ADOs. A measurable quantity is obtained by tracing over the bath, which is equivalent to the selection of solely the reduced density operator from the hierarchy of ADOs. Similarly, all ADOs can be obtained by applying creation or annihilation operators to adjacent ADOs forming a basis in the HEOM space,^59^ similar in contents to a vibrational eigenstate basis. Even though physical information from this HEOM space basis is not directly accessible by a measurement, the system–bath coupling encoded on the ADOs manifests itself by their connections within the hierarchy, which involves feedback to the reduced density matrix.^25,60^ The Matsubara space is a purely mathematical construction, but each dimension relates to both the physical characteristics and the magnitude of the Markovian constraint applied. In this hierarchy, we require the sum of an infinite number of Matsubara frequencies where the first two frequencies (ν_*n*0_ and ν_*n*1_) are temperature-independent and related to the bath dissipation rate, and subsequent thermal frequencies (ν_*nl*_), which are complex, result from the poles in the spectral function.
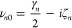
10
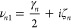
11
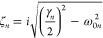
12
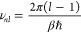
13

**Figure 1:**
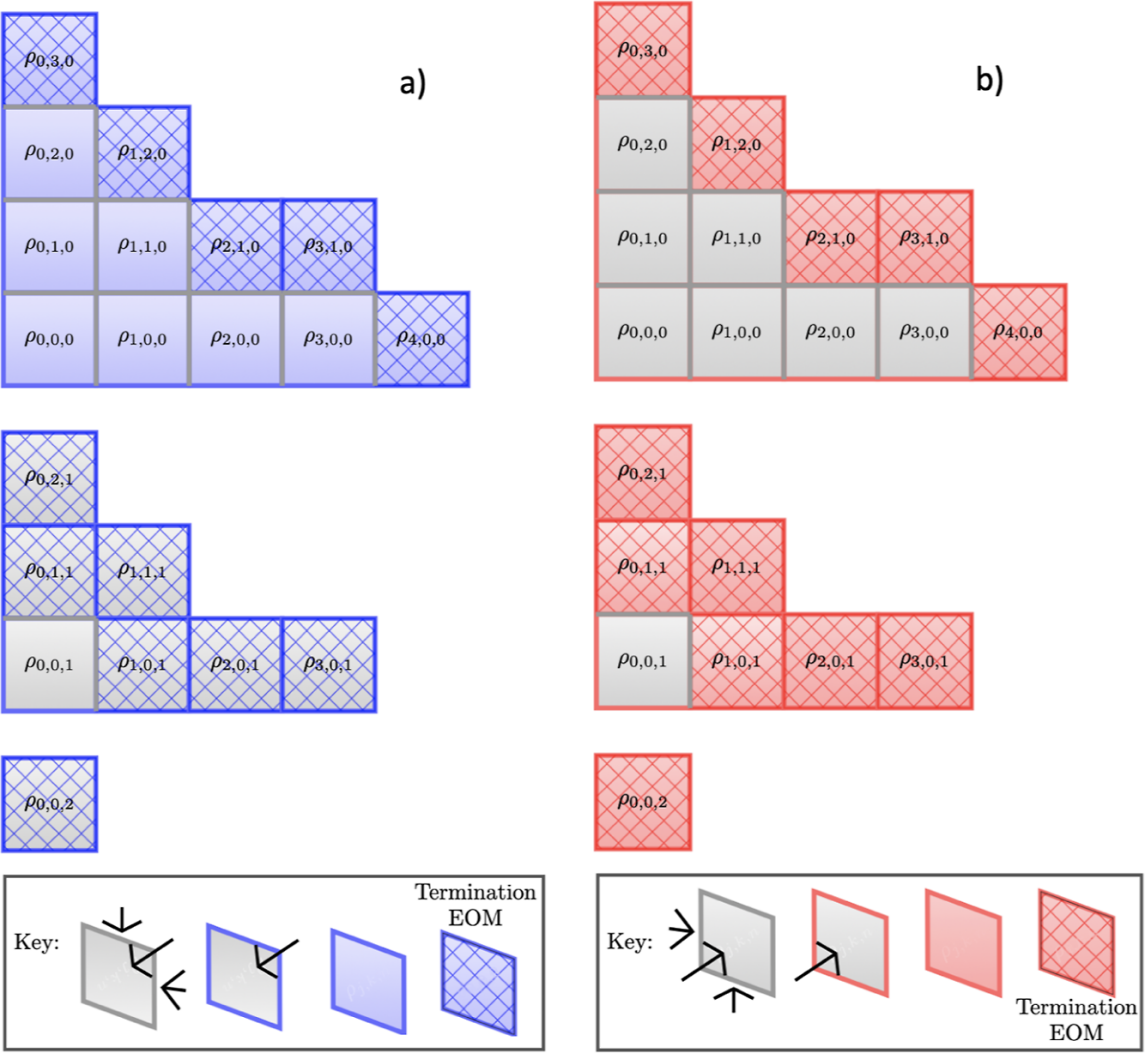
Idealized underdamped hierarchy structure. The freedom of information to flow upward or downward is depicted with blue and red colors, respectively. Specifically, a blue boundary signifies that information can enter this ADO from ADOs in the plane with a lower *z* value. Red boundaries signify that information can enter this ADO from ADOs in the plane with a larger *z* value.

As such, the Markovian constraint, Γ_max_, applied on each ADO determines the number of auxiliaries and the total number of Matsubara dimensions where each frequency becomes its own independent axis.
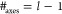
14
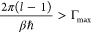
15Figure 1 depicts an idealized underdamped hierarchy structure with three Matsubara axes (*x*, *y*, *z*) to be generalized below. In reality, these structures would be much more complex, generally with dimensions >3, particularly for structured spectral densities. Within this diagram (a) presents the movement of information away from the density matrix, ρ_0,0,0_, with each subplot representing two-dimensional slices across the *xy*-plane for a different value of *z*. Similarly, (b) shows information movement back toward the density matrix. Each face of the cubes which represent the ADOs are colored based on whether they permit a transfer of virtual information in that particular direction. In addition, the cross-hatching of some ADOs denotes that they are governed by the alternative terminating equation of motion and will be unable to transfer non-Markovian virtual information beyond this boundary. In the discussion that follows, we refer to ADOs increasing in tier as they move out along a Matsubara axis, away from the density matrix.

Describing a Matsubara coordinate concisely is difficult due to the (usual) high dimensionality of a Matsubara space. In order to discuss individual ADOs, we need to define each of the axes and positions along those axes. We can define each Matsubara dimension as an integer *M*{·} and the ADO position along that axis as *n*. From this definition, we could, for example, write an ADO which is tier 3 in *M*1 and tier 2 in *M*5, but zero along all other axes, within an eight-dimensional Matsubara space as (3,0,0,0,2,0,0,0), or more concisely as 3_1_2_5_.

In Figure 1, each ADO has up to six possible quantum channels. The left half of Figure 1 shows where information can move outward, increasing in tier, from 0_0_. The blue faces indicate that information cannot move back to the *M*1*M*2 (*xy*) plane from *M*3 (*z*). Similarly, information cannot move out of 2_3_ as it is a terminator. The right side of Figure 1 indicates where information moves back toward the density matrix, decreasing in tier, toward 0_0_. The red face in 2_3_ denotes that it is a terminator and that information cannot move down from higher tiers, and the red plane *M*1*M*2 at *M*3 tier 0 indicates that information cannot move down beyond *M*3 tier 0 (i.e., *n*_tot_ ≥ 0). This can be written generally as *n*_*m*_, where *n* is the tier of a specific Matsubara dimension and *m* is the index of that dimension, e.g., *m* = 1 ≡ *M*1. The total tier, or distance measure, of an individual ADO vector is written as *∑n* = *n*_tot_. In this notation, it is important to note the distinction between those Matsubara frequencies which are explicit and temperature-independent (ν_*n*0_ and ν_*n*1_) and those which are part of the infinite sum of temperature-dependent frequencies (ν_*nl*_), particularly when the system is underdamped. The lowest Matsubara dimensions (one for an overdamped system, and two for underdamped systems) will be temperature-independent and associated with undamped vibrational oscillations, and all remaining frequencies relate to the influence of damping on this mode. Consequently, the temperature-independent frequencies exhibit profoundly non-Markovian dynamics, but the temperature-dependent frequencies do not.

At this point, it is pertinent to comment on the mathematical structure of the ADOs and how they may be interpreted physically. While information flow between individual auxiliaries cannot be measured experimentally, the specific structure of the Matsubara space containing the ADOs nevertheless dictates the nature of the system–bath coupling and therefore the actual dynamics of the specific OQS. The trace distance is a purely mathematical metric used to compare two different matrices of the same type (i.e., those occupying a common Hilbert space). Typically, these measures are used to quantify information flow with reference to real density matrices (those describing a measurable state) and specifically the direction of information flow between a system and a bath in an OQS.

While ADOs do not represent measurable quantum states, information contained within the bath is real, as it is exchanged with the system. It is therefore useful to talk about the exchange of phonons between ADOs, within the bath, and this can be tracked theoretically via the trace distance metric. In order to emphasize the fact that this information is not directly measurable and does not constitute a useful resource from the point-of-view of quantum information theory, we refer to this as virtual information.

The nature of the auxiliaries and their intrinsic relation to the system parameters are discussed by Fay et al.^43^ and Yan et al.,^61^ but the level of physical insight that can be gained from the auxiliaries is not discussed. In contrast, authors such as Zhu et al.^60^ propose an explicit system–bath correlation by creating a collective bath coordinate from the auxiliaries. Additionally, Xing et al.^62^ produce an imaginary time HEOM and use this to explicitly calculate real correlation functions through the path integral formalism. Both of these methods are impactful studies of the ADOs, but both craft bespoke operations to analyze the auxiliaries; neither consider the transfer of information between ADOs and how this might be used.

In this study, we consider a vibronic molecule with a fundamental vibration subsumed into the spectral density to systematically analyze how this impacts information flow within the ADOs. This is linked directly to electronic spectral broadening. From this, we aim to ascertain the form and magnitude of information flow through the ADOs. We term this information virtual information in order to abstract it from the physical quantum information within the density matrix, the former being a mathematical tool and the latter an experimentally observable quantity. We analyze and characterize the behaviors within the ADOs with respect to their Matsubara coordinate via the BLP metric. From this, we draw conclusions to optimize the computational efficiency for OQS dynamics in general and apply this specifically to two-dimensional electronic spectroscopy (2DES) simulations. We propose a new hierarchy termination constraint based on the BLP metric which is useful when modeling vibronic molecules within the condensed phase.

## 3 Methodology

### 3.1 System Hamiltonian

The model is constructed as a two-level electronic monomer with ground and excited electronic states, |*g*⟩ and |*e*⟩, with a fundamental transition frequency of . Each electronic level has a set of *N* vibrational states with the vibrational frequency . As described below, these are produced by canonically transforming the single vibrational mode into the environment ensemble of phonon modes.^63^ The harmonic potentials for the electronic states result in a monomer system Hamiltonian
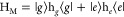
16where the nuclear constituents for the ground and excited electronic states are
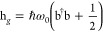
17
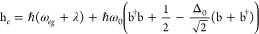
18respectively, where b^(†)^ is the lowering (raising) operator.^64^ Here, dissipation is defined as the relaxation of the system excitation energy through coupling to environmental degrees of freedom. Dephasing occurs as a direct consequence of environmentally induced stochastic perturbations driving the system’s potential energy surface away from equilibrium. This manifests physically as a decoherence of wavepackets. A reorganization energy, , is induced through the displacement of the excited-state potential, with respect to the ground-state minimum, along the nuclear coordinate by Δ_0_. This generates the monomer Hamiltonian.

### 3.2 Open-System Model

From the system Hamiltonians, an OQS can be constructed through coupling to the environment spectral density. This makes use of the HEOM—which is intrinsically dependent on its spectral function—the details of which can be found in our previous work^54,65^ and in the original derivation by Tanimura and Kubo.^14^

All environmental degrees of freedom, including all memory effects due to non-Markovianity, are described by the correlation function associated with the fluctuation–dissipation theorem
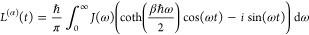
19where β = (*k*_B_*T*)^−1^. Within 19 dissipation terms arise from the sine-dependent terms, whereas the thermally induced fluctuations are a consequence of the cosine terms.

Our model is a reduction from the full vibrational structure of the Hamiltonian, resulting in only the essential electronic structure being explicit. To achieve this, the fundamental intramolecular vibrational mode from the vibronic monomer system of interest is subsumed into the bath degrees of freedom through a canonical transform. The remaining electronic states are then coupled to an underdamped (U) Brownian oscillator and spectral density.
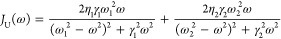
20

The two components of the spectral density correspond to the intramolecular vibrational mode in the underdamped limit,^66^ ω_1_ ≫ γ_1_, such that ω_1_ = ω_0_ and η_1_ = λ, and the bath modes, respectively. In the overdamped limit, where ω_2_ ≪ γ_2_, the second contribution is reduced to the Debye form
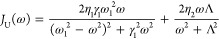
21where
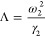
22

In contrast to the skewed Gaussian profile of the overdamped spectral density, the underdamped spectral density features a sharp Lorentzian peak at the intramolecular mode frequency, with width determined by the damping parameter γ_1_. For this approach, an underdamped HEOM is derived from the multicomponent spectral density, resulting in a multicomponent HEOM.^67^ Note that when the vibration is subsumed into the bath, γ_1_ introduces additional damping which is not present when the vibration is contained in the Hamiltonian. Approaching the limit γ_1_ → 0, the two approaches become equivalent, as discussed within ref (65), but in practice some additional damping from the second bath is unavoidable.

With this model, we are able to generate 2D electronic spectra^68−71^ in the impulsive limit using the response function formalism, as described in the appendix of ref (54).

### 3.3 Regimes of the Simulations

In the following set of simulations, we consider three cases which we label the overdamped (fastest dissipation rate), intermediate, and underdamped (slowest dissipation rate). We note that although the mathematical form of the bath is underdamped, all cases can be constructed by the consideration of the damping parameters. In all simulated 2D spectra, we use a ground- and excited-state separation of . The vibrational mode of the system is , where the (dimensionless) displacement of the excited-state potential is Δ_0_ = 1.09, giving a reorganization energy of λ̃ = 300 cm^–1^. The parameters in the case of the overdamped regime (ODR) are γ̃_1_ = 1750 cm^–1^ and γ̃_2_ = 2500 cm^–1^ such that Λ̃ = 100 cm^–1^. The intermediate damping regime (IDR) case has γ̃_1_ = 100 cm^–1^ and γ̃_2_ = 2500 cm^–1^ such that Λ̃ = 100 cm^–1^. The underdamped regime (UDR) case has γ̃_1_ = 100 cm^–1^ and γ̃_2_ = 300 cm^–1^. In all cases, the bath coupling and vibrational mode frequency are η̃_1_ = λ, ω_1_ = ω_0_, η̃_2_ = 50 cm^–1^, and . The simulations are performed at 300 K, within the bounds of the high-temperature approximation, and the Markovian limit Γ_max_ = 2000 cm^–1^. The HEOM simulations for the ODR, IDR, and UDR models contain 1264, 39149, and 98513 ADOs, respectively. Two-dimensional spectra are generated with a coherence time up to τ = 200 fs in steps of 0.5 fs, for population times of *T* = 0, 100, and 200 fs.

The two series of states for which the trace distance are calculated, eq 2, correspond to one which has been excited by interaction with a laser of fwhm 20 fs and one which has not.^54^

## 4 Results and Discussion

### 4.1 Virtual Information Flow

**Figure 2:**
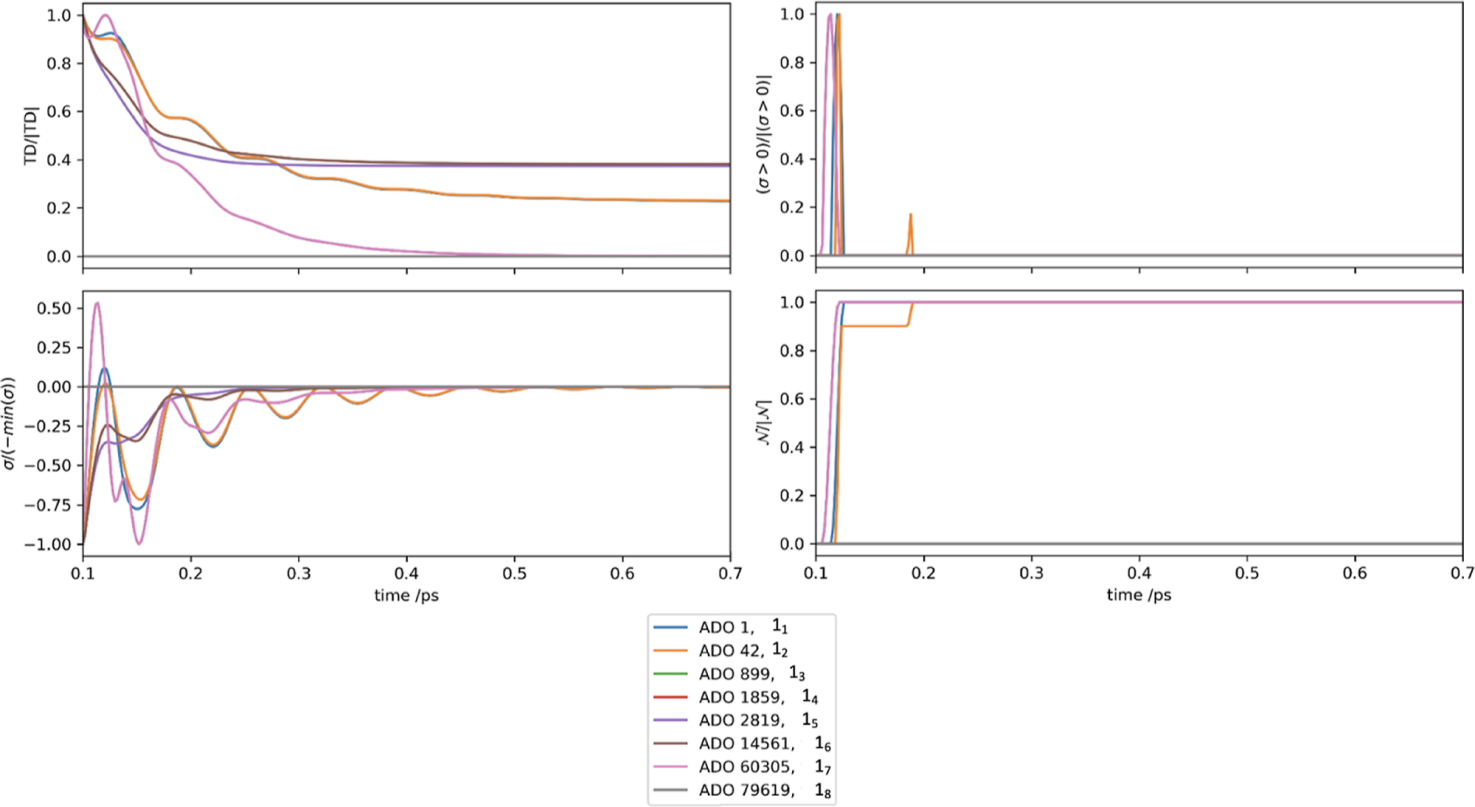
UDR case (with  cm^–1^ and  cm^–1^) showing (a) normalized trace distance, (b) normalized positive flux, (c) normalized flux, and (d) normalized BLP measure for 1_{·}_.

Figure 2 presents all ADOs where *n* = 1 for the UDR case. This hierarchy contains eight Matsubara dimensions, so the ADOs considered are {1_1_, 1_2_, ..., 1_8_}. All of these axes have similar trace distance profiles; however, it is clear from the total virtual information flux that each axis has a unique behavior. The smaller Matsubara frequencies, 1_1_ and 1_2_, initially have a maximum Markovian transfer of virtual information, followed by a recurrence of virtual information on approximately 40% that of magnitude, within the first 100 fs, whereas the ADOs in higher Matsubara dimensions, 1_7_ and 1_8_, have a high non-Markovian feedback before they become Markovian with a predominantly monotonic loss of information. The fact that the total information flux (c), positive information flux (b), and resultant BLP metric (d) are so different for each case suggests that the role of the different Matsubara axes in contributing to the bath dynamics is unique.

**Figure 3:**
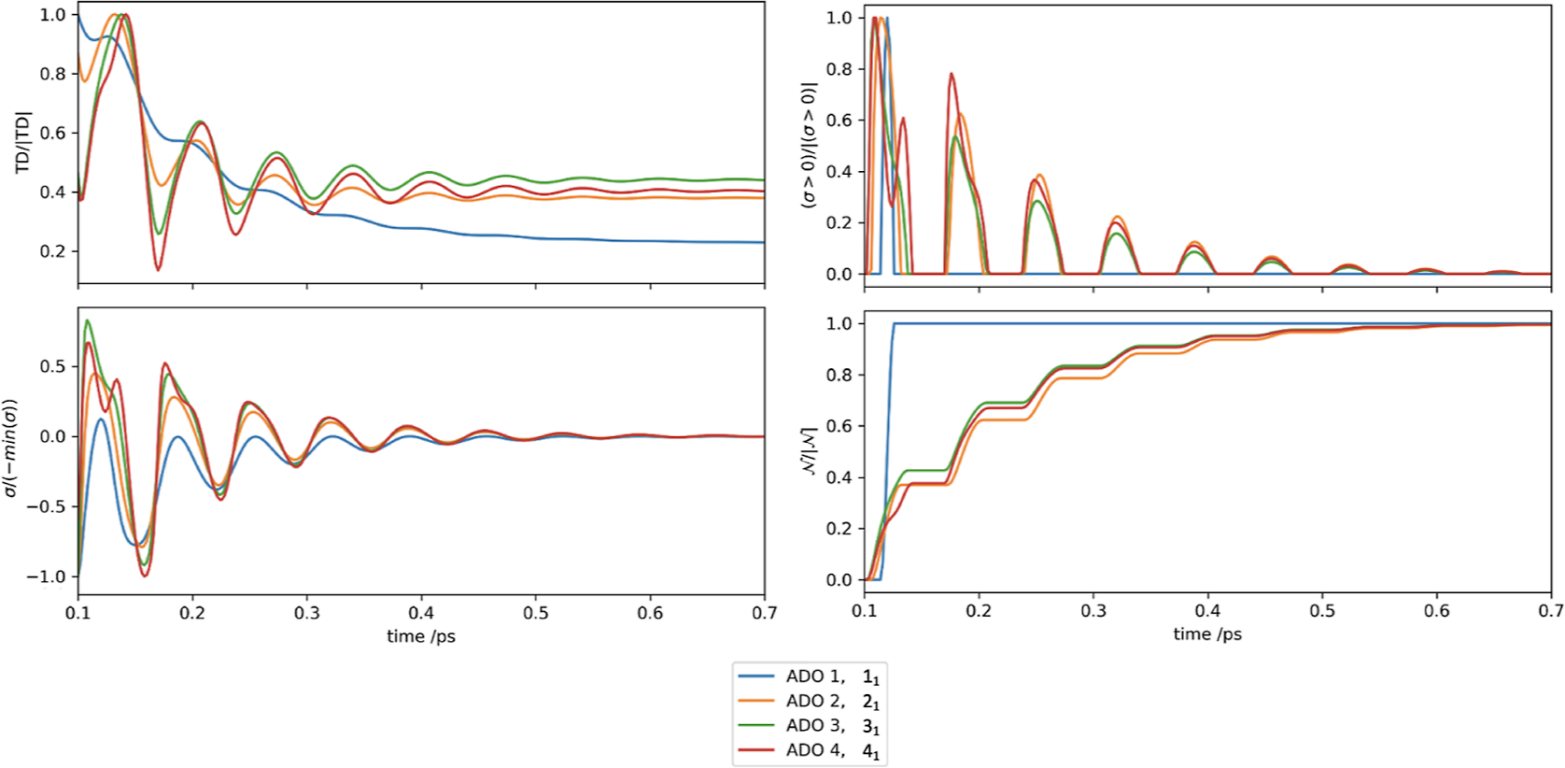
UDR case ( cm^–1^ and  cm^–1^) showing (a) normalized trace distance, (b) normalized positive flux, (c) normalized flux, and (d) normalized BLP measure for *n*_1_, *n* = {1, 2, 3, 4}.

In direct contrast to this, Figure 3 presents contributions of consecutive ADOs from the Matsubara axis, *M*1, {1_1_, 2_1_, 3_1_, 4_1_}. All four subplots show very uniform oscillating patterns with small changes in amplitude based on the position of the ADO along the axis. There is a linear decrease in the equilibrium value of the trace distance as a result of the normalized positive virtual information flux having sharper oscillations and a steeper gradient. Based on the uniformity of oscillations and peak locations in the positive flux, it is clear that information flow through ADOs in the same axis is very similar, whereas ADOs of the same tier but in different Matsubara axes are quite different. The linear increase in the relative peak amplitude also highlights that higher tier ADOs are accompanied by a corresponding increase in virtual information recurrence. This does not mean that higher tier ADOs contribute more virtual information but that relative to their size each tier contributes a larger proportion of the maximum virtual information content.

These findings can be generalized further based on the analogous results from the FDR and IDR regimes. Despite their smaller ADO numbers, the same trends exist. Namely, ADOs from the same Matsubara axis behave in a similar fashion, but auxiliaries of an equivalent tier within different axes have little in common. Furthermore, we consider whether this behavior can be extrapolated to multitier ADOs (i.e., ADOs with *n* > 0 across multiple axes) or some share character. Results from the multitier analysis indicate that, similar to the results in Figures 2 and 3, ADOs are most similar to others in their own Matsubara axis, and all axes are largely independent of each other.^72^

Next, we consider the magnitude of virtual information contained within the ADOs as a function of time. Since the information content of ADOs decreases in amplitude proportionally with their tier position, the vast majority of auxiliaries contain very little virtual information. For clarity, we consider the first 200 ADOs and note that these should contain a large percentage of the total virtual information. The aim of this analysis is to determine whether there are some ADOs of greater intrinsic importance to the OQS dynamics relative to others.

**Figure 4:**
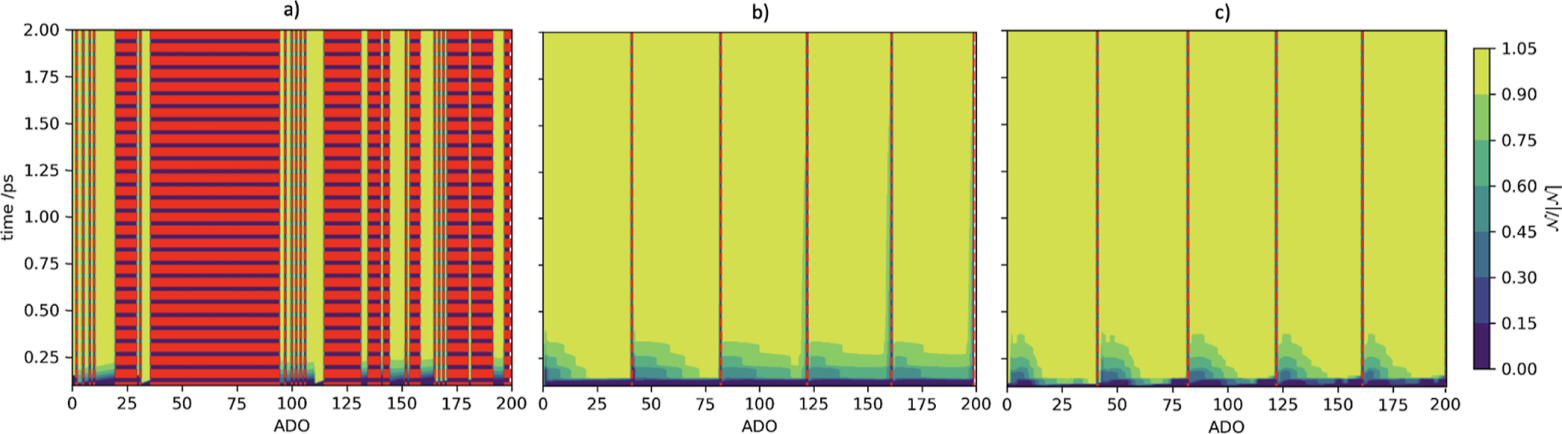
Contour plot of time, ADO, and BLP metric for (a) ODR, (b) IDR, and (c) UDR regimes of the model system. Red dashed lines denote terminators.

Figure 4 presents contour plots for the change of the BLP metric through time for the first 200 ADOs, for the ODR and the UDR cases. The order of the first 200 ADOs is dependent on the specific structure of the HEOM implementation; however, this is equivalent to including those ADOs contained by *n*_1_ ≤ 37, *n*_2_ ≤ 4, and *n*_R_ = 0, where R = {3, 4, 5, 6, 7, 8}, tiers bound. The vertical red lines depict which auxiliaries are terminators. In the UDR regime, there are very few terminators in the first 200 ADOs, with each terminator representing the end of a different Matsubara axis. Additionally, there is a structure in the BLP for early ADOs, with each Matsubara axis contributing to recurrence to the system from the bath. Within these regions, the BLP is the lowest in the first few hundred femtoseconds for the lowest ADO numbers. This corresponds to the recurrence of virtual information feeding back toward the density matrix most rapidly for lower ADOs and tiers. This is consistent with the fact that higher tiers correspond to thermal Markovian behaviors. Additionally, UDR relative to IDR lacks the well-defined recurrence structure at early times. This clearly correlates with the fact that recurrence of information occurs much more readily in underdamped cases, compared to overdamped scenarios.

**Figure 5:**
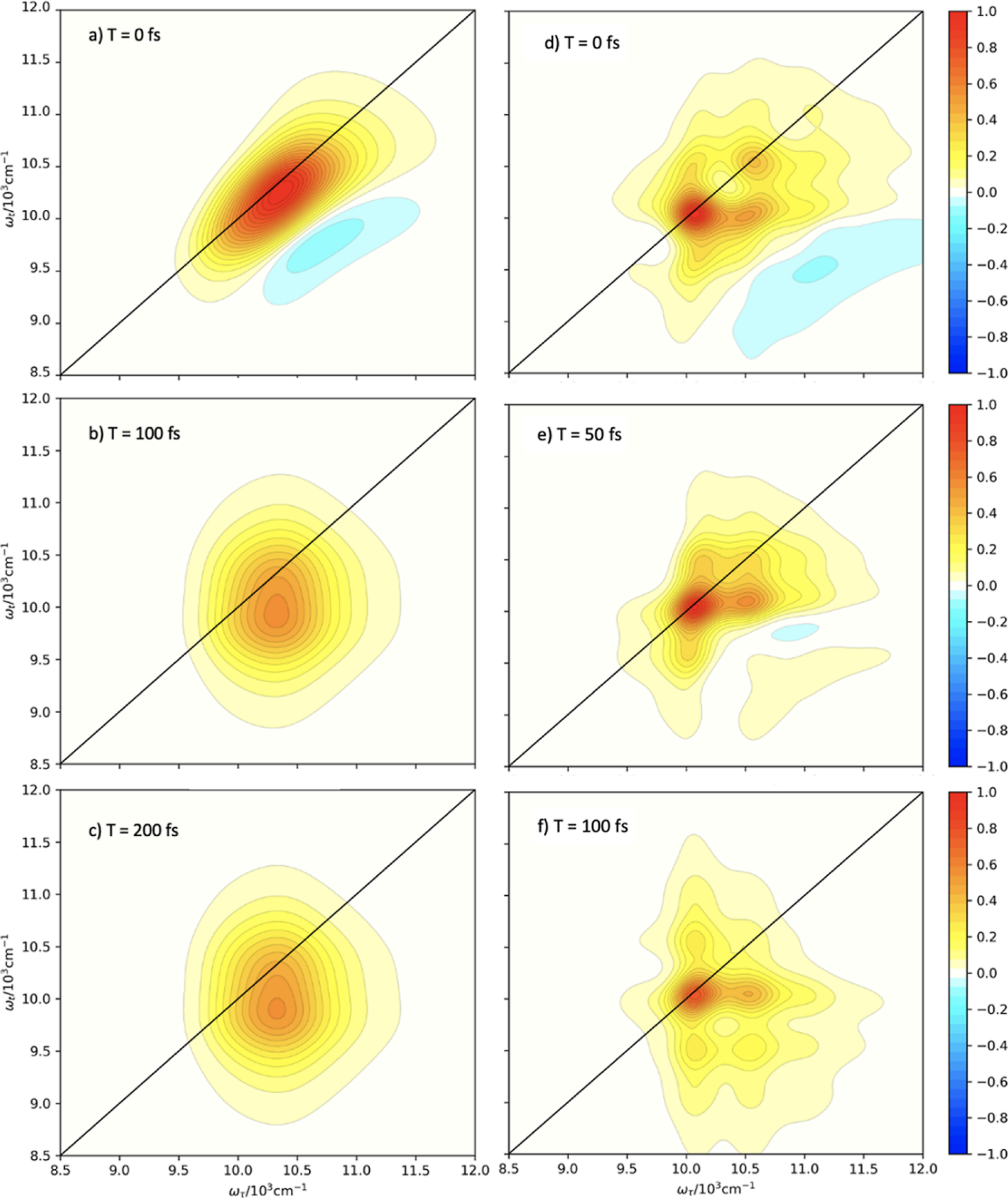
Two-dimensional electronic spectra for ODR (left), IDR (center), and UDR (right) regimes of the model system at *T* = 0, 100, and 200 fs.

We also consider 2D electronic spectroscopy for the UDR and ODR regimes. Figure 5(a–c) presents spectra for the ODR case for 0, 100, and 200 fs. The peak position is obscured by the very large inhomogeneous broadening introduced by the fast vibrational mode that is subsumed into the environment. This broadening results in what appears to be a single large peak, although the intensity differences at ∼ (10300, 10300) cm^–1^ for (a) and ∼ (10300, 9750) cm^–1^ for (b,c) suggest that there is a large Stokes shift in which the vibrational character and population relax into the ground state. Clearly, a large number of terminators, relative to the total ADO number, is associated with a significant increase in inhomogeneous broadening. In contrast, in the UDR case, (g–i) the spectra have very precise positions, and many cross-peaks are present. Even at low times, the peaks are Lorentzian in shape, with a mixture of inhomogeneous and homogeneous broadening as a consequence of the underdamping of the modes being subsumed into the spectral density. This results in high positional precision and low broadening accuracy due to a low number of terminators and a more uniform spread of relative virtual BLP over all of the ADOs.

From these results, it is clear that there is some redundancy with the behavior of individual auxiliaries along a specific Matsubara axis. For example, in Figure 3, we observe that *n*_1_, *n* = {1, 2, 3, 4} behave in an almost identical fashion such that this characteristic is being replicated four times within the hierarchy. These results present an opportunity for us to develop a new strategy for terminating the ADO hierarchy. The original termination approach used in these simulations, found in (ref (73)), is that developed by Dijkstra and Prokhorenko.^73^ Now, we consider a new set of trajectories that also exploit a new termination which effectively reduces the number of auxiliaries based on the redundant behavior presented above. We consider the termination of ADOs that possess similar character to others but with the smallest amount of virtual information content, that is, those with higher ADO numbers within a Matsubara axis. Continuing the example of *n*_1_ for *n* = {1, 2, 3, 4}, the new termination scheme would reduce this to *n*_1_ for *n* = {1, 2}, where *n* = {3, 4} would now become terminators.

By canonically subsuming a pure intramolecular vibrational mode as an underdamped vibration, additional damping is added to the model. This is referred to as canonically derived damping or canonical damping. The additional termination scheme based on this damping is therefore termed canonically derived/canonical termination.

### 4.2 Impact of Canonically Derived Termination

**Figure 6:**
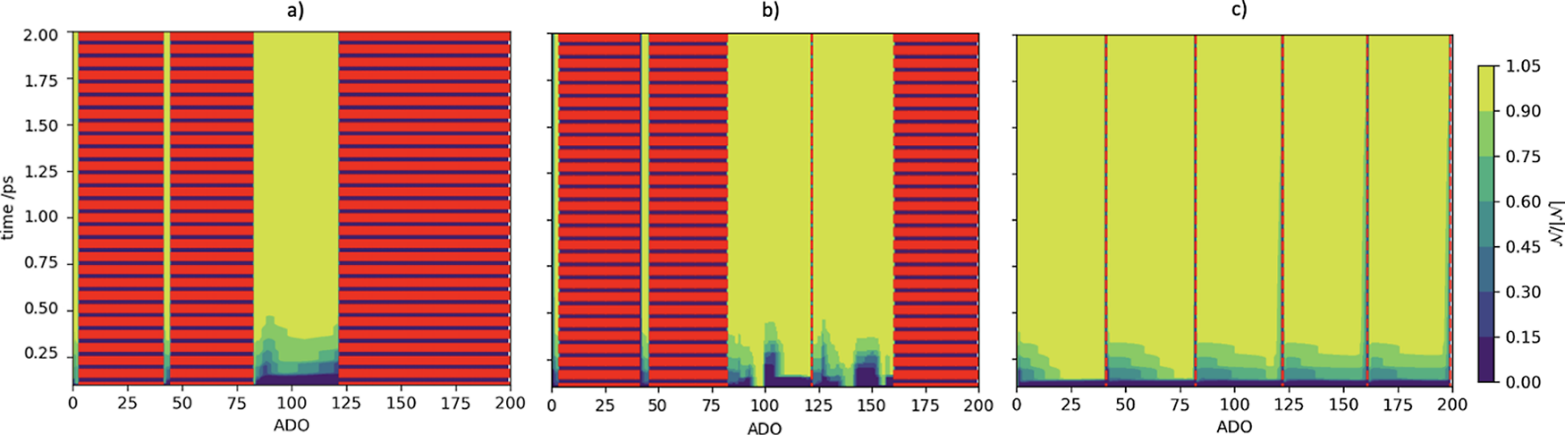
Contour plot of time, ADO, and BLP metric for the IDR model (a) with termination *n*_{·}_ > 2, (b) with termination *n*_{·}_ > 3, and (c) without canonical termination. Red dashed lines denote terminators.

We test the proposed secondary termination scheme by considering two cases: termination of ADOs *n*_{·}_ > 2 and *n*_{·}_ > 3, in order to demonstrate a convergence toward the original BVM hierarchy. By construction, this scheme will tend to terminate thermal Matsubara axes preferentially over the nonthermal ones, as they necessarily contain more non-Markovian virtual information relating to the system vibration. Such schemes significantly reduce the section of the hierarchy in which information can flow back to the density matrix and consequently constitute a computational saving. Figure 6 shows contour plots of the BLP measures for the IDR case in each of the termination regimes. It is clear from comparing Figure 6(a–c) that this system behaves very similarly to the UDR case in Figure 4c however, the virtual information recurrence at an early time is more pronounced based on the reduced damping, leading to decreased damping. From Figure 6a, when *n*_{·}_ > 2, it can be seen that the first 75 ADOs go in sequence along the first two Matsubara dimensions. The third block (the next ∼50 ADOs) is multitiered and therefore is not terminated by the secondary criterion. These therefore contribute significantly to the OQS dynamics. It is clear that the majority of ADOs are terminated, in a manner similar to the ODR regime, so we might expect equivalent levels of broadening in the resultant 2DES. Additionally, in Figure 6b, when *n*_{·}_ > 3, it is clear that another set of multitier ADOs remains as a consequence of the increased cutoff in the second termination, as is evident in the two unterminated regions between ADOs ∼75 and ∼150 which are separated by a single terminated ADO. As the value of *n*_tot_ increases, the number of dimensions included, and consequently the number of multiphonon processes included, increases up to the full IDR in Figure 6c.

**Figure 7:**
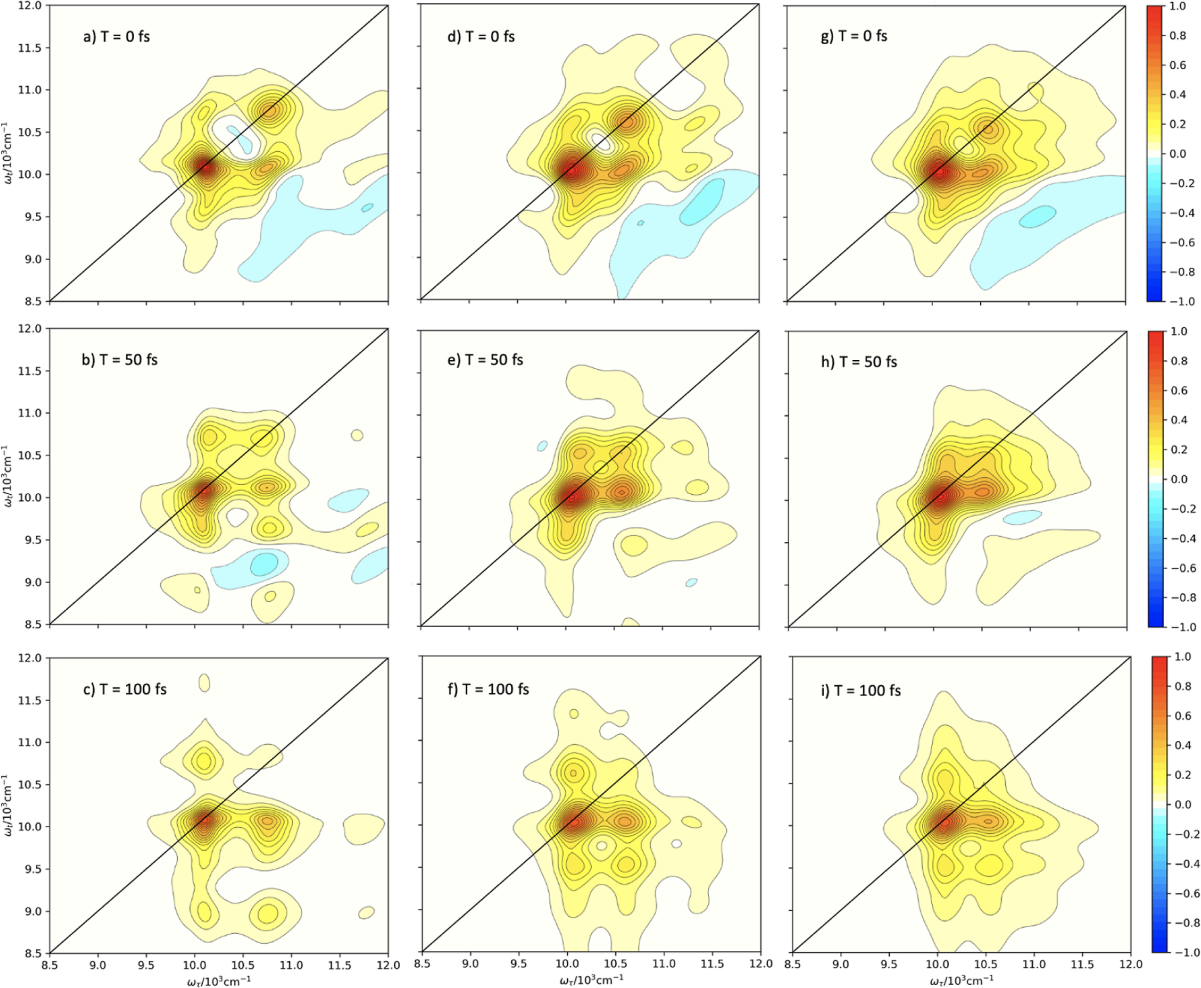
2D electronic spectra for the IDR with an *n*_{·}_ > 2 canonical termination (a–c), with an *n*_{·}_ > 3 canonical termination (d–f), and without canonical termination (g–i) at *T* = 0, 50, and 100 fs.

The spectra in Figure 7 show the corresponding 2DES associated with the respective plots in Figure 6. We note that the spectra in Figure 7(g–i) are exactly those in the study by Humphries et al.,^65^ which employs the same model with the IDR parameters. They show a reasonable level of inhomogeneous broadening as a consequence of the subsumed vibrational mode, but vibronic peaks are still clearly distinguishable. All peaks, including the cross-peaks, are broadened individually by the coupling to the bath, resulting in more broadening than in the UDR case but significantly more peak precision than in the ODR regime.

In comparison, the spectra in panels (a–f) show qualitatively similar peak profiles. Despite a significant percentage of the total hierarchy being terminated, the individual peaks have well-resolved positions and have not been overbroadened. As demonstrated by Figure 5 (a–c), regimes which are far into the overdamped limit tend to produce hugely overbroadened peaks with very minimal positional resolution, but this is not evident after canonical termination. In contrast, the peaks have clear Lorentzian character, and additional peaks have become apparent, examples being at approximately (10000, 11750), (11750, 10000), and (11750, 9000) wavenumbers in Figure 7c.

In order to test the quality of the truncated hierarchies, we generate difference spectra for the termination of *n*_{·}_ > 2 and *n*_{·}_ > 3 with respect to the full IDR, in the absence of canonical termination. We then integrate over their absolute values to form a quantification of the total broadening difference as we progress from the full number of ADOs and truncate the hierarchy.

**Figure 8:**
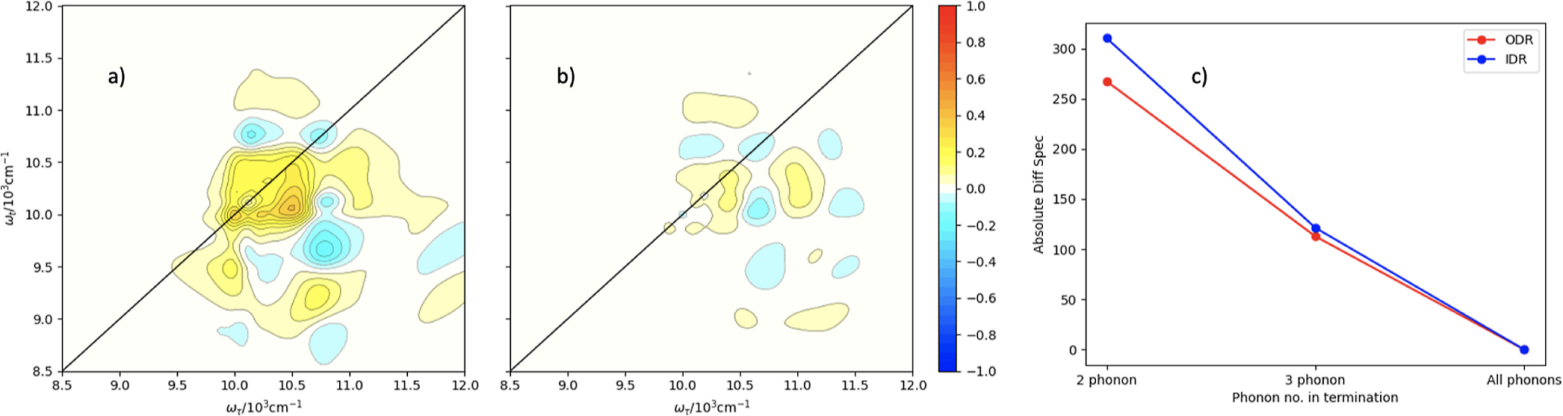
(a) Difference spectrum for IDR-terminated to include up to two phonon processes subtracted from the IDR with all phonons. (b) Difference spectrum for IDR-terminated to include up to three phonon processes subtracted from the IDR with all phonons. (c) Integrated absolute difference spectrum metric plotted for each of the three termination regimes for both the ODR and IDR damping regimes.

Figure 8(a,b) shows the two difference spectra for the IDR spectrum in Figure 7(g) minus Figure 7(a,d), respectively. Panel (c) shows the integrated absolute value for each of the spectra. It is clear from the negative correlation shown in this figure that there is a linear improvement in the accuracy of the model as *n* increases toward the full number of phonon processes. Terminating two phonon processes can be quantified as having an area difference of ∼300 cm^–2^ and that changing the termination to three phonon processes halves this area to ∼150 cm^–2^.

**Figure 9:**
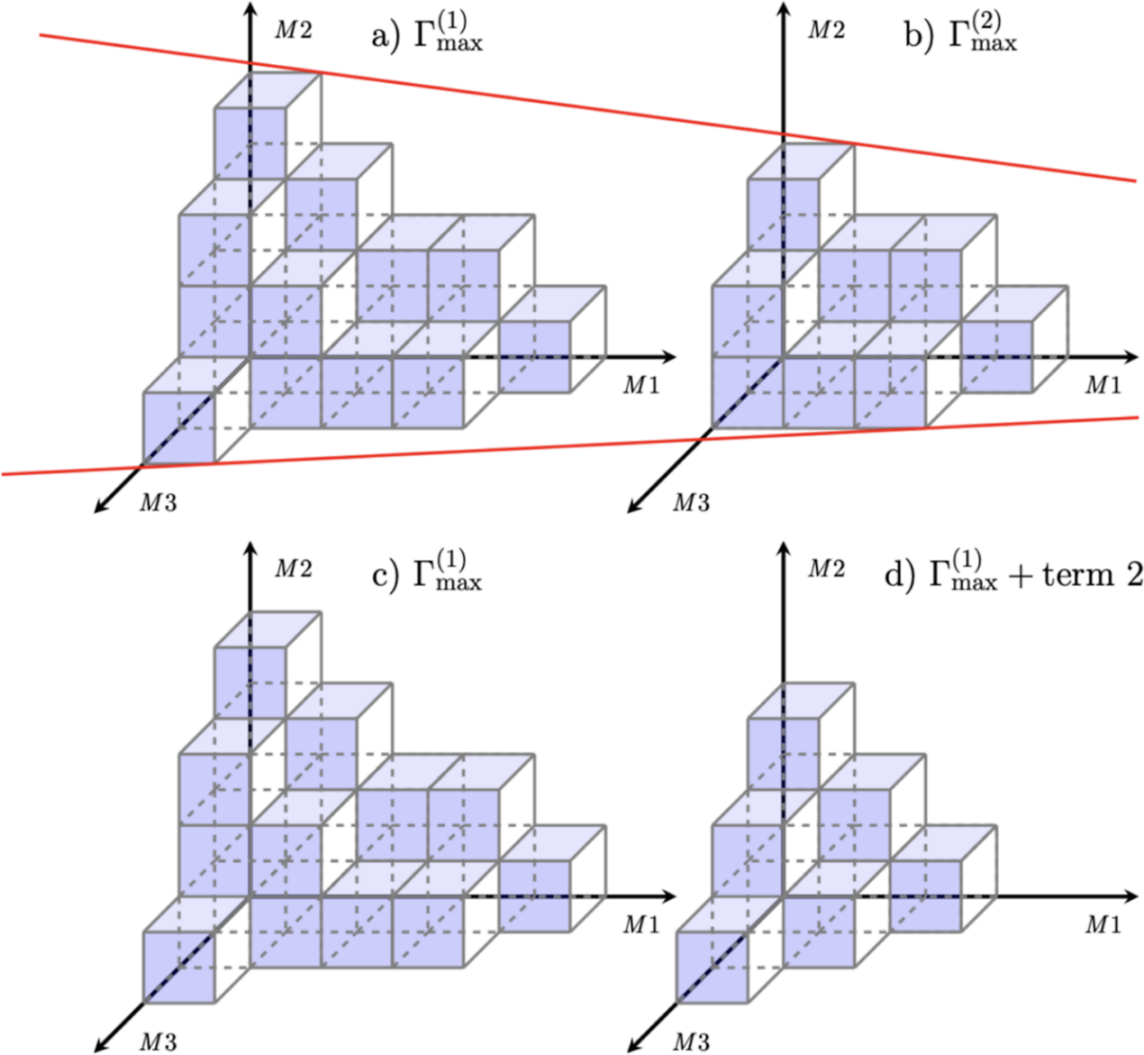
Schematic showing the closed hierarchy volume. (a,c) Arbitrary value of Γ_max_^(1)^. (b) For Γ_max_^(2)^ < Γ_max_^(1)^. (d) First Γ_max_^(1)^ and then the secondary termination scheme. Movement from (a,b) demonstrates a self-similar volume, whereas (c,d) demonstrates a regime without this restriction.

Therefore, the most significant virtual information is stored within the lowest tier ADOs. This gives a clear indication that, in specific parameter regimes, it would be feasible and useful to truncate the hierarchy at different ADOs along different axes, thereby reducing its overall volume. Using the original termination criteria, eq 9, the hierarchy is a sealed volume which can only be reduced by decreasing Γ_max_, resulting in a hierarchy of smaller volume but similar shape. However, we propose that it may be possible to optimize the HEOM for specific cases by setting different lengths for each Matsubara axis. This could maximize the accuracy of the simulated spectra relative to the experimental spectra while reducing the hierarchy volume and hence reducing the simulation time with a minimal impact on the quality of the spectra. The hierarchy reduction is demonstrated pictorially in Figure 9.

## 5 Conclusions

The HEOM are an infinite system of equations that evolve individual ADOs. The equations are coupled via raising and lowering operators that allow the movement of phonons between ADOs. Once truncated, the resulting ADO structures can take on a variety of shapes with varying complexity. Within these structures, ADOs are represented as elements lying along independent Matsubara axes, with their positions along the axes being referred to as tiers. In this work, we show that ADOs contain virtual information whose flux through the hierarchy can be monitored. This can provide insight into the relative importance of individual ADOs in defining the bath. Crucially, the new termination scheme intrinsically accounts for the temperature dependence of each Matsubara axis upon termination. Therefore, ADOs dependent on high, temperature-dependent dimensions are preferentially terminated over those which are of low dimensions and necessarily more non-Markovian. A qualitative understanding about the nature of the system–bath interaction can therefore be obtained directly from the virtual information metric BLP measure when it is applied to the ADOs. We have clearly demonstrated differing recurrence times of virtual information for ADOs across the hierarchy as a consequence of non-Markovian feedback. From this, we can gain insight into the resulting impact on the spectral broadening and peak precision in 2DES. Finally, we employed a termination scheme based on the physical understanding afforded by this analysis. The resulting spectra from the canonical termination procedure are in qualitative agreement with those produced using the original hierarchy, thereby reducing the computational cost for 2D spectra of OQS with complex baths. We quantify this as being a difference of ∼300 cm^–2^ in total broadening for up to two phonon processes, which is halved (resulting in twice as good an approximation) for three phonon processes. Future work will involve optimizing the approach for the development of a new termination procedure for systems with strong non-Markovian feedback.

## Data Availability

Key data are available from zenodo,^74^ and all other data can be regenerated from the appropriate code upon reasonable request to the corresponding authors.
